# CRISPR/Cas9 technology for advancements in cancer immunotherapy: from uncovering regulatory mechanisms to therapeutic applications

**DOI:** 10.1186/s40164-024-00570-y

**Published:** 2024-10-19

**Authors:** Xiaohang Feng, Zhengxing Li, Yuping Liu, Di Chen, Zhuolong Zhou

**Affiliations:** 1 Department of Colorectal Surgery, the Second Affiliated Hospital, and Zhejiang University-University of Edinburgh Institute, Zhejiang University School of Medicine, Zhejiang University, Hangzhou, China; 2 Biomedical Sciences, College of Medicine and Veterinary Medicine, Edinburgh Medical School, The University of Edinburgh, Edinburgh, UK; 3 Center for Reproductive Medicine of The Second Affiliated Hospital, Zhejiang University School of Medicine, Zhejiang University, Hangzhou, Zhejiang China

**Keywords:** CRISPR/Cas9, Genome editing, Immune checkpoint inhibitor, CAR-T cell therapy, Immune invasion

## Abstract

In recent years, immunotherapy has developed rapidly as a new field of tumour therapy. However, the efficacy of tumour immunotherapy is not satisfactory due to the immune evasion mechanism of tumour cells, induction of immunosuppressive tumour microenvironment (TME), and reduction of antigen delivery, etc. CRISPR/Cas9 gene editing technology can accurately modify immune and tumour cells in tumours, and improve the efficacy of immunotherapy by targeting immune checkpoint molecules and immune regulatory genes, which has led to the great development and application. In current clinical trials, there are still many obstacles to the application of CRISPR/Cas9 in tumour immunotherapy, such as ensuring the accuracy and safety of gene editing, overcoming overreactive immune responses, and solving the challenges of in vivo drug delivery. Here we provide a systematic review on the application of CRISPR/Cas9 in tumour therapy to address the above existing problems. We focus on CRISPR/Cas9 screening and identification of immunomodulatory genes, targeting of immune checkpoint molecules, manipulation of immunomodulators, enhancement of tumour-specific antigen presentation and modulation of immune cell function. Second, we also highlight preclinical studies of CRISPR/Cas9 in animal models and various delivery systems, and evaluate the efficacy and safety of CRISPR/Cas9 technology in tumour immunotherapy. Finally, potential synergistic approaches for combining CRISPR/Cas9 knockdown with other immunotherapies are presented. This study underscores the transformative potential of CRISPR/Cas9 to reshape the landscape of tumour immunotherapy and provide insights into novel therapeutic strategies for cancer patients.

## Introduction

Traditional clinical approaches for cancer therapy include resection surgery [1], chemotherapy [2] and radiotherapy [3]. These treatments are ineffective in completely eliminating metastatic tumours, which have a high tendency to reoccur [4]. They fail to address the problem of tumour cell migration, which can lead to metastasis and the recurrence of lesions [5]. In addition, these treatments are linked to many adverse effects, including radiation-induced harm and drug toxicity [6]. Immunotherapy offers a safer alternative to the aforementioned methods [7].

Immunotherapy seeks to activate or restore the cytotoxicity of the body's immune response to tumours by initiating an active immune response or by enhancing the efficacy of existing immune cells [8]. Several immunotherapies have demonstrated clinical efficacy, including cancer vaccines [9], antibody drug conjugates (ADCs) [10], immune checkpoint blockade [11] and cellular therapies such as chimeric antigen receptor (CAR) therapy [12]. The development of tumour vaccines has yielded promising outcomes in certain forms of cancer, including advanced prostate cancer and melanoma. However, tumour vaccines still face challenges of immature preparation technology and the selection of appropriate antigens. The efficacy of these therapies in a wider range of cancer types has not met expectations, as evidenced by the results of clinical trials [13, 14]. Many ADCs have demonstrated significant efficacy in clinical trials and have consequently been granted regulatory approval to treat various types of cancer. For example, T-DM1(trastuzumab emtansine) is approved for use in patients with HER2-positive breast cancer. Nevertheless, the field of ADCs is still struggling to address systemic toxicity and resistance while maintaining the stability of ADCs and reducing immune-related adverse reactions [15]. Immune checkpoint inhibitors, including those targeting PD-1/PD-L1 and CTLA-4, have achieved remarkable results in many solid tumours and haematological malignancies. Furthermore, clinical trials investigating their potential are also actively underway [16]. However, the regimen of these immune checkpoint inhibitors remains challenged by acquired resistance, the risk of adverse immune events, and a lack of effectiveness [17]. Additionally, PD-1/PD-L1 is the only predictive biomarker for these therapies, making detection results less accurate in the presence of interference of the complex tumour environment [18]. CAR-T cell therapy has shown remarkable efficacy in the treatment of B-cell lymphoma, leukemia, and multiple myeloma, and it has been approved for use in several countries around the world [12]. However, due to the complex immunosuppressive microenvironment of solid tumours, the efficacy of CAR-T in treating solid tumours is significantly diminished. In addition, the severe side effects of CAR-T therapy, including cytokine release syndrome (CRS) and immune effector cell-associated neurotoxic syndrome (ICANS), require further investigation [19].

While immunotherapy has shown positive effects in patients, its efficacy is still limited by the intricate nature of tumours and underlying immune escape mechanisms [20]. Precise CRISPR/Cas9 gene editing technology provides a powerful tool to overcome the limitations of existing immunotherapies and to improve the safety and effectiveness of various treatments. CRISPR/Cas9 technology can induce the knockout of multiple inhibitory molecules (such as PD-1, CTLA-4, and LAG-3) in T cells, thereby enhancing the expansion and persistence of CAR-T cells. CAR-T cells optimized by CRISPR/Cas9 technology are able to overcome resistance mechanisms within T cells and in the tumour microenvironment [21]. By modifying specific genes, CRISPR/Cas9 can help tumour cells be more easily recognized and attacked by the immune system. This approach has the potential to develop more effective and targeted tumour immunotherapy products, such as improving the immunogenicity of personalized tumour vaccines, or reducing the toxicity of ADCs [9, 22].

CRISPR/Cas9 gene editing technology allows for the precise manipulation of the human genome by selectively inserting or disrupting specific genes [23]. The method has been widely used in the generation of animal tumour models, the investigation of regulatory mechanisms of drug resistance, the exploration of epigenetic regulation, and the development of innovative tumour therapeutics. CRISPR/Cas9 technology can precisely edit and modify the genes of tumour cells and immune cells to reduce immunosuppressive effects and enhance immune system function. This can significantly improve the effectiveness and safety of immunotherapy, showing great prospects in tumour immunotherapy.

This study introduces the existing cancer immunotherapies that are being used and explores the potential application of genome-wide CRISPR/Cas9 knockout technology in tumour immunotherapy. The following section provides a concise overview of both preclinical and clinical investigations that have employed CRISPR/Cas9 technology in tumour immunotherapy. Finally, we explore possible collaborative methods and innovative applications of CRISPR/Cas9 knockout technologies in conjunction with other immunotherapies, as well as their potential impact and prospects for enhancing tumour immunotherapy.

### Rationale for CRISPR/Cas9 technology

#### Explanation of the CRISPR/Cas9 system and its components

**Fig. 1:**
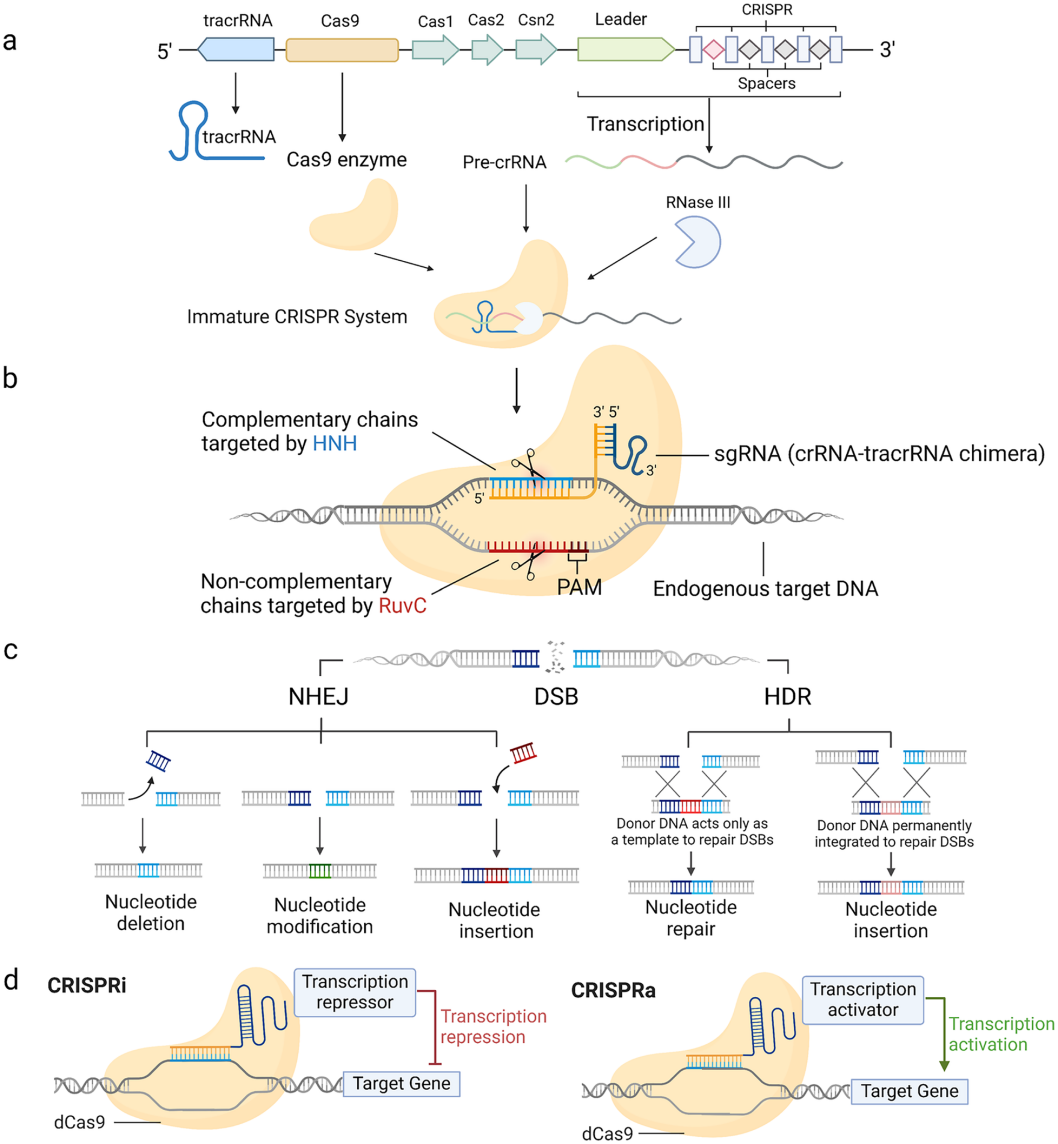
CRISPR/Cas9 system gene editing principles, and the derived CRISPRi and CRISPRa. **a** The basic structure of CRISPR/Cas9 loci is illustrated; **b** A mature CRISPR/Cas9 system, formed by sgRNA and Cas9 proteins, exhibits the ability to cleave target DNA; **c** Cells repair double-strand breaks (DSBs) through either non-homologous end joining (NHEJ) or homology-directed repair (HDR) pathways; **d** Cas9 enzyme gives rise to two genome-manipulation tools known as CRISPRi and CRISPRa

The CRISPR/Cas9 system functions as a set of genetic "scissors" capable of cleaving double-stranded DNA [24]. The CRISPR/Cas9 system consists of a single guide RNA (sgRNA) and a Cas9 protein as an endonucrenase [25]. SgRNAs consist of CRISPR RNA (crRNA) and trans-activated crRNA (tracrRNA) paired by complementary bases, with the help of an external enzyme, RNase III [26, 27] (Fig. 1a). The main function of it is to guide the Cas9 protein to make precise cuts on the target DNA sequence [26, 28]. When Cas9 binds to the target sequence, it makes a double-strand break (DSB) approximately three bases upstream of the Protruding Associated Motif (PAM) site [29]. This cleavage is facilitated by Cas9 's two catalytic domains: the HNH domain, which cuts the complementary DNA strand, and the RuvC domain, which cuts the non-complementary strand (Fig. 1b).

#### Mechanisms of CRISPR/Cas9-mediated gene knockout

CRISPR/Cas9 knockout technology is primarily derived from the cellular method of self-healing DSBs, which occasionally leads to the introduction of mutations during DNA repair. Cellular non-homologous end-joining (NHEJ) or Homology-mediated repair (HDR) machinery is responsible for repairing these brakes which can potentially lead to errors that cause alterations in gene sequences. This mechanism allows targeted inactivation of specific genes [30].

In laboratory settings, NHEJ has superseded end-joining and single-stranded heat treatment as the predominant DNA repair process. The process relies on the use of homologous templates to reconnect DNA termination sites [31]. NHEJ is a process that introduces minor genetic insertions, deletions, or substitutions during DNA break repair. These alterations can lead to mutations in the genetic code, causing disruptions in gene function. HDR is another alternative repair route that can be used. In its natural context, HDR typically relies on sister chromatids as repair templates. However, for gene editing and knock-in applications, it necessitates the use of an external donor [32] (Fig. 1c). The efficiency of NHEJ and HDR varies greatly by cell cycle stage and cell type. HDR is a relatively high-fidelity repair method that is commonly used for gene knock-in. It is only more effective in the S and G2 phases of the mammalian cell cycle, especially in embryonic stem cells. Because during these cycle stages, the presence of homologous DNA templates within the cell promotes HDR progression. NHEJ is active throughout the cell cycle. At the same time, it is the preferred route to repair DNA breaks when the requirement for repair speed takes precedence over accuracy [33].

The ability of Cas9 to be programmed allows for a wider range of options for gene editing tools. Scientists can modify a homologous Cas9 protein variant, rendering it inactive in both structural domains. This modified protein, known as dCas9, is an enzyme that lacks catalytic activity and can only attach itself to the designated target site. This inhibits the transcription of the specific gene, thereby effectively reducing gene expression. Derivatives of CRISPR, CRISPRi and CRISPRa allow for more precise genomic control by combining transcriptional regulators or epigenetic modifiers to activate or suppress expression of target genes (Fig. 1d**)**. CRISPRi hinders RNA polymerase activity and facilitates DNA methylation or targeted RNA alterations, leading to gene suppression. CRISPRa, on the other hand, boosts the activity of a particular gene by using dCas9, which increases the expression of that gene and facilitates epigenetic modifications to activate gene function.

#### Advantages and limitations of CRISPR/Cas9 over other gene editing technologies

Currently, the most effective genome editing tools are those based on the following technologies: meganucleases, zinc finger nucleases (ZFN), transcription activator-like effector nucleases (TALEN) and CRISPR/Cas9 [34–36]. Comparisons of the four gene editing technologies are shown in Table 1. CRISPR/Cas9 technology is free from the high degree of customization of DNA recognition proteins, and its guide RNA (gRNA) design and synthesis effort are much less than the construction of recognition modules for TALEN and ZFN technologies. In CRISPR/Cas9 technology, Jinek’s team covalently joined crRNA and tracrRNA to create a single guide RNA, ensuring accurate targeting for cleavage [26]. Compared to custom proteins, custom RNAs are easier to manipulate and more economical. However, the system still exhibits certain limitations. One of the main problems is the prevalence of off-target effects (OTEs) [37]. Unexpected double-stranded breaks triggered by off-target cutting can lead to mutations that impair cellular gene function.

**Table 1:** Comparison of four gene editing techniques

Techniques	Meganuclease	ZFN	TALEN	CRISPR/Cas9
Gene editing system				
Target site size	12–40 bp	9 or 18 bp	14–20 bp	About 20 bp + NGG
Cleavage site	Determined by endonuclease	Between two ZFN binding sites	Between two TALE binding sites	Near PAM sequence
Endonuclease	I-CreI, I-SceI etc	FokI	FokI	Cas9
Design and cost	Complicated/high cost	Complicated/high cost	Complicated/high cost	Simple/low cost
Editing efficiency	Low	Low	Low	High
Multilocus editing	Customize multiple proteins	Customize multiple proteins	Customize multiple proteins	Using multiple sgRNAs
Advantage	High specificity and low cytotoxicity	Mature platform	Simpler and more specific than ZFN	Low miss rate, cheap and easy
Limitation	Intracellular delivery is difficult and limited application range	High off-target rate and cytotoxicity	The module assembly is complicated, large sequencing workload	Can not be cut without PAM, and the specificity is not high

### Application of CRISPR/Cas9 technology in tumour immunotherapy

#### Screening and identification of immunomodulatory genes by CRISPR/Cas9

**Fig. 2:**
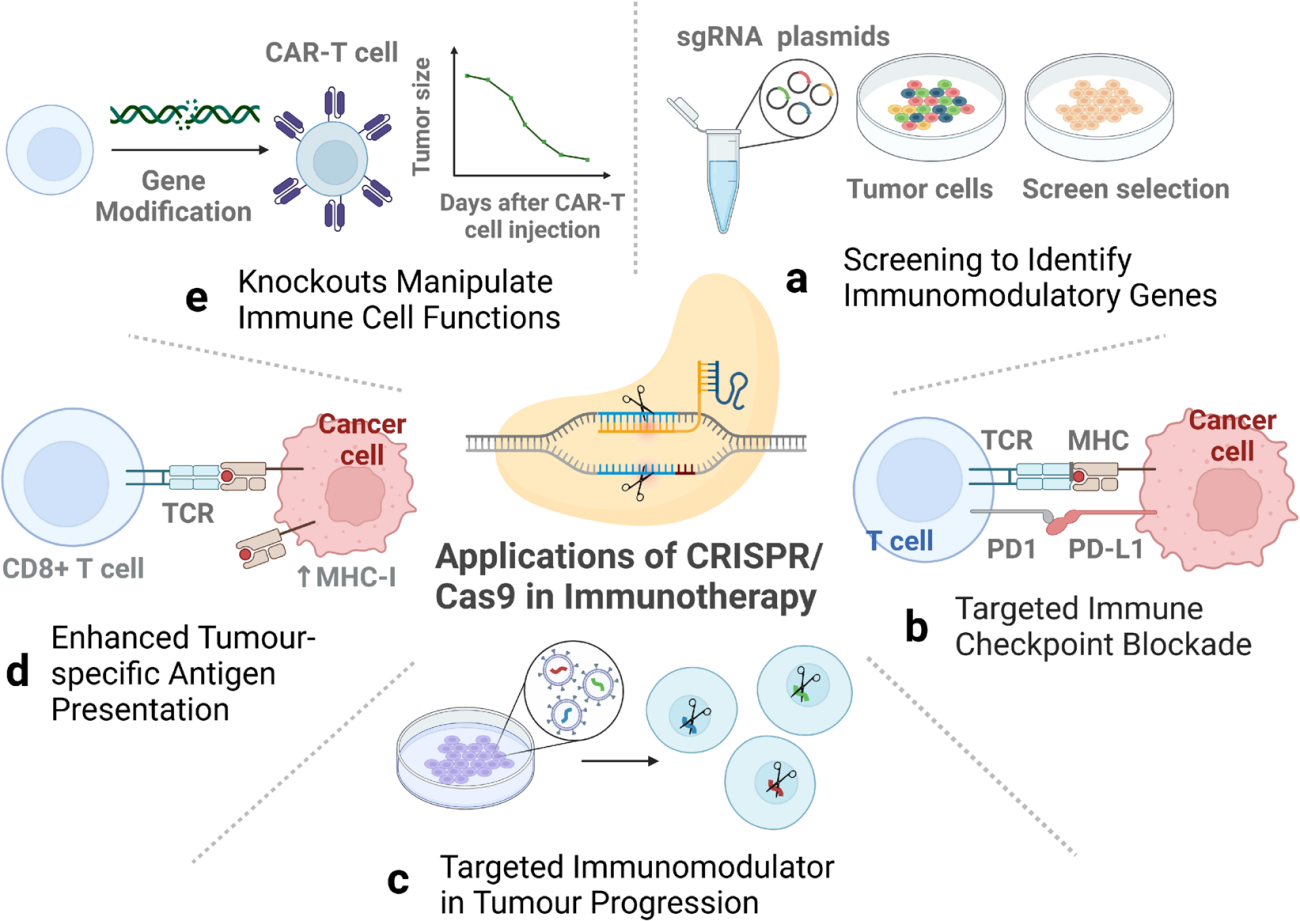
CRISPR/Cas9 technology identifies and accurately screens for genes with immunomodulatory functions, facilitating the selective knockout or knock-in of genes. CRISPR/Cas9 knockouts can block these checkpoint pathways to activate the immune system against cancer cells. The technology can also target and destroy genes involved in tumour immune evasion mechanisms such as key immunosuppressive factors in tumour cells. CRISPR/Cas9 knockout upregulates genes associated with MHC class I molecules, thereby enhancing tumour-specific antigen presentation. CPISPR/Cas9 technology performs precise genetic modification of engineered T cells for optimization of immune cell function

The application of CRISPR/as9 screening technology has facilitated the precise identification and selection of genes with regulatory immune functions at the genome-wide level. If a gene shows potential utility by modulating the tumour microenvironment or enhancing the ability of immune cells to attack tumour, then targeting the gene will lead to innovative breakthroughs in tumour immunotherapy (Fig. 2a). Manguso's team designed a specific sgRNA library that was targeted for in vivo knockout screening of immunomodulatory genes in a mouse tumour model. The library targets 2368 genes expressed in melanoma cells, with the potential to reveal previously unidentified immunotherapy targets [38]. This study not only confirmed the roles of known immune escape molecules such as PD-L1 and CD47, but also revealed that deletion of the IFN-γ signalling pathway may lead to resistance to immunotherapy. Among them, protein tyrosine phosphatase (PTPN2) was found to be activated by IFN-γ signalling and could be a new potential target for cancer immunotherapy. In another study, Dong et al. found that knockdown of the DEAH-Box Helicase 37 (DHX37) gene can enhance the response of CD8 + T cells to breast cancer following an in vivo CRISPR screening on CD8 + T cells [39]. Ye et al. developed a CRISPR activation screen based on dead guide RNA (dgRNA) in primary CD8 + T cells to identify gain-of-function (GOF) targets for CAR-T cell engineering. Targeted knock-in or overexpression of PRODH2, significantly enhanced the killing capacity and in vivo efficacy of CAR-T cells in multiple cancer models. In addition, transcriptomic and metabolomic analyses revealed that enhanced PRODH2 expression could affect metabolic pathways and improve mitochondrial and immune functions of CAR-T cells [40]. Matthew et al. identified CD19 regulators (ZNF143 and NUDT21) in B-cell progenitor acute lymphoblastic leukaemia using genome-wide CRISPR/Cas9 screening techniques. ZNF143 activates the promoter of CD19 and promotes transcription. However, NUDT21 inhibited the expression of CD19 mRNA [41]. By performing genomic CRISPR/Cas9 loss-of-function screening, Olli et al. found that the death receptor signalling of FADD and TRAIL-R2 is key to participation in anti-CD19 CAR-T cell therapy. These signals affect the ability of CAR T cells to kill CD19-positive tumour cells. The results of the screening data provide a theoretical basis for optimising anti-CD19 CAR T cell therapy [42]. In a genome-wide CRISPR/Cas9 screening, Ludivine et al. confirmed that the MS4A1 gene encodes CD20, which is the target of rituximab. At the same time, they found that interferon regulatory factor 8 (IRF8) can influence anti-CD20 antibody-induced cytotoxicity and phagocytosis [43]. The application of CRISPR/Cas9 screening technology can reveal key genes related to immunotherapy and their regulatory mechanisms. This makes it a valuable tool for improving the accuracy of cancer immunotherapy.

#### Targeting immune checkpoint molecules by CRISPR/Cas9 technology

Tumour immune checkpoints are responsible for regulating immune responses, maintaining self-tolerance, and preventing autoimmune reactions. The tumour cells in cancer employ checkpoints to inhibit the immune system's assault, thereby facilitating immune evasion. Immune checkpoint inhibitors activate T cell attack on tumours by removing this inhibition. Therefore, they are considered to be the key drugs in current cancer therapies. FDA-approved immune checkpoint inhibitors include Ipilimumab (Yervoy, CTLA-4 antibody), Nivolumab (Opdivo, PD-1 antibody), Pembrolizumab (Keytrude, PD-1 antibody), and Atezolizumab (Tecentriq, PD-L1 antibody). The use of CRISPR/Cas9 technology to block the checkpoint activation pathway has been shown to enhance the effect of tumour immunotherapy (Fig. 2b).

Engineered immune cells generated using CRISPR knockout technology can be used as a universal immunotherapy for cancer or in combination with CAR-T engineering to enhance the efficacy of T cells in eliminating cancer cells. CRISPR/Cas9 technology can directly knock down the PD-1 gene in primary T cells. This results in an increased stimulation of T cell activation by dendritic cells, which in turn effectively enhances the anticancer toxicity of T cells [44].

Similarly, PD-1 can be knocked down in cytotoxic T lymphocytes (CTLs), which inhibit regulatory T cell (Treg) activity and reduce their number, thereby recruiting more effector cells [45, 46]. Yang et al. managed to specifically knockdown the PD-L1 and PTPN2 genes in tumour cells, attenuating inhibition of the JAK/STAT pathway, which in turn further amplified CD8 + T cell susceptibility to tumours [47]. Lu et al. also developed a natural polymer-based plasmid delivery vector to knockout the β-catenin gene for the purpose of inducing downregulation of PD-L1 expression [48].

Scientists have found that loss-of-function of the RNA-specific adenosine deaminase ADAR1 in tumour cells enhances immunotherapy sensitivity and overcomes resistance to immune checkpoint blockade [49]. In this study, loss of ADAR1 triggers double-stranded RNA ligand sensing through PKR and MDA5, leading to tumour growth inhibition and inflammation. Thus, the loss-of-function of ADAR1 overcomes resistance to PD-1 checkpoint blockade. In addition, it was found that induction of sufficient inflammation in interferon-sensitive tumours bypasses the need for CD8 + T cell recognition, providing a general strategy to overcome immunotherapy resistance. Sang's study revealed that receptor-interacting protein kinase 2 (RIPK2) plays a pivotal role in immune evasion in T cell-mediated cytotoxicity in pancreatic cancer. Targeting RIPK2 genetically or pharmacologically could enhance the sensitivity of pancreatic ductal adenocarcinoma (PDAC) to anti-PD-1 immunotherapy, resulting in prolonged survival or complete remission. This study provides a rationale for novel combination therapies of RIPK2 inhibitors with anti-PD-1 immunotherapy [50].

#### Targeting immunomodulator in tumour progression by CRISPR/Cas9

Disruption of genes involved in tumour immune escape represents a further avenue of tumour immunotherapy, as it impairs the ability of tumour cells to use these genes to evade immune system attack. When these key genes are disrupted or inhibited, tumour cells become more susceptible to immune system recognition and destruction, thereby improving the efficacy of treatment (Fig. 2c). Frangieh et al. developed the Perturb-CITE-seq technique to explore the innate immune checkpoint inhibitor (ICI) resistance mechanism of cancer cells. This high-throughput technique combines perturbation of the CRISPR-Cas9 gene with single-cell transcriptome (RNA expression) and protein analysis. In a co-culture model of patient-derived melanoma cells and autologous tumour-infiltrating lymphocytes (TILs), the study identified known and novel mechanisms of immune resistance, including defects in the interferon-gamma (IFN-γ)-JAK/STAT signalling pathway and the antigen presentation pathway, as well as the loss/down-regulation of CD58 [51].

While performing a CRISPR screen on a mouse model of lung cancer, Dubrot's team identified the immune regulator Adam2 as a cancer antigen that inhibits tumour antigen presentation and suppresses interferon expression [52]. This finding potentially explains why cytotoxic T cells exhibit more potent cytotoxic effects in Adam2-overexpressing tumours following their in vitro expansion and self-metastasis. Non-small cell lung cancer (NSCLC) frequently involves mutations in the KRAS gene, and there is a clear need for more clinically accessible targeted therapies that specifically target the KRAS downstream signalling pathway. In a transplantable KRAS-mutant lung cancer model, researchers examined an innovative in vivo CRISPR screening technique and identified adenosine synthase COX-2 as an immune regulator [53]. COX-2 expression is driven by KRAS. The results indicate that reducing COX-2 may enhance the effectiveness of immunotherapy for KRAS-mutant NSCLC by targeting KRAS-mediated immune evasion mechanisms.

#### Enhancement of tumour-specific antigen presentation by CRISPR/Cas9

The effectiveness of immune checkpoint blockade therapies is often limited. Enhancing tumour antigen presentation could be a promising therapeutic strategy (Fig. 2d). The major histocompatibility complex (MHC) on the surface of tumour cells binds to antigenic peptides, presenting the antigen on the cell surface and being recognized by immune system T cells. Downregulation of MHC molecule expression leads to immunosuppression, or the escape of cancer cells from immune surveillance. Zhou's team found that CRISPR-mediated inhibition of EZH2 was able to upregulate MHC class I expression and CD8 + T-cell proliferation in head and neck squamous cell carcinoma (HNSCC) [54]. This could significantly improve cancer cell killing with PD-1 therapy. Abnormal delivery of MHC-I antigen in acute myeloid leukaemia (AML) leads to immune evasion of T cells. To investigate this resistance mechanism, Chen used specific CRISPR/Cas9-induced peptide-MHC-I screening process to identify potential therapeutic targets in an AML model, identifying three key MHC-I negative regulators, surface protein 6 (SUSD6), transmembrane protein 127 (TMEM127), and E3 ubiquitin ligase WWP2 [55]. Using a combination of CRISPR, proteomics, and transcriptomics mechanism experiments, Marta Canel et al. showed that FAK signaling loss increased expression of the immunoproteasome and major histocompatibility complex I (MHC-I), enhancing antigenic diversity and presentation. This regulatory role of FAK is independent of its kinase activity but requires FAK nuclear translocation. In addition, this study also reveals new strategies that may enhance the efficacy of pancreatic cancer therapy by degrading FAK [56].

Conversely, knockout of key MHC-I antigen-presenting genes, such as B2M, TAP1, TAP2, and HLA, facilitates the ability of cancer cells to avoid being destroyed by CD8 + T cells and NK cells. Significant gene deletions include HLA, B2M, TAP1, TAP2, and TAPBP. These genes also impact the functionality of the IFN-γ and EIF2 signalling pathways [57]. In a CRISPR screen performed by Shifrut et al., it was found that the capacity of T cells to eradicate cancer cells was markedly diminished when the expression of LCP2, a positive regulator of TCR signalling, was knocked down in A375 cells. However, the knockdown of negative regulators, such as TCEB2, SOCS1, CBLB and RASA2, greatly enhanced the immune-killing capabilities of T cells [58]. Using a genome-wide CRISPR/Cas9 screen, Andrews found that the TCR recognizes and destroys cancer-specific cells through the antigen-presenting molecule MR1 (MHC class I-associated protein 1) and does not recognise recognise non-cancer cells. This recognition is achieved by sensing cancer metabolites [59]. This study investigates and confirms that MR1 possesses the ability to recognise recognise T cell receptors (TCRs) in several types of cancer cells. This finding provides novel insights into the development of HLA-independent, pan-cancer, and pan-population T cell immunotherapy.

#### Manipulation of immune cell function by CRISPR technology

CPISPR/Cas9 technology can accurately modify the genes of immune cells. CRISPR knockout on human immune cells successfully manipulates the intricate immune regulatory network in the tumour microenvironment (TME). To illustrate, it enhances the effectiveness of chimeric antigen receptor-engineered T cell immunotherapy (Fig. 2e). This platform enables the efficient manufacture of therapeutically modified T cells on a massive scale, which can be customized for more complex clinical treatment approaches. CAR-T therapy allows the genetic modification of T cells in a laboratory setting, enabling them to identify and destroy tumour cells without relying on HLA. Scientists used CRISPR/Cas9 technology to interfere with PD-1 to enhance the activity of generic CAR-T cells targeting EGFRvIII in glioma [60]. It was found that edited CAR-T cells produced pro-inflammatory cytokines more efficiently and showed enhanced anti-tumour activity in vitro. Furthermore, in an animal model, these gene-edited CAR-T cells, delivered intravenously or intracerebroventricularly, significantly prolonged survival and effectively controlled tumours in mice. This suggests that CRISPR/Cas9 gene editing may enhance CAR-T cell therapy against gliomas by overcoming PD-L1-mediated immunosuppression [61].

### Preclinical and clinical studies of tumour immunotherapy Using CRISPR/Cas9 knockout technology

#### Overview of preclinical studies examining CRISPR/Cas9 knockout in animal models

**Fig. 3:**
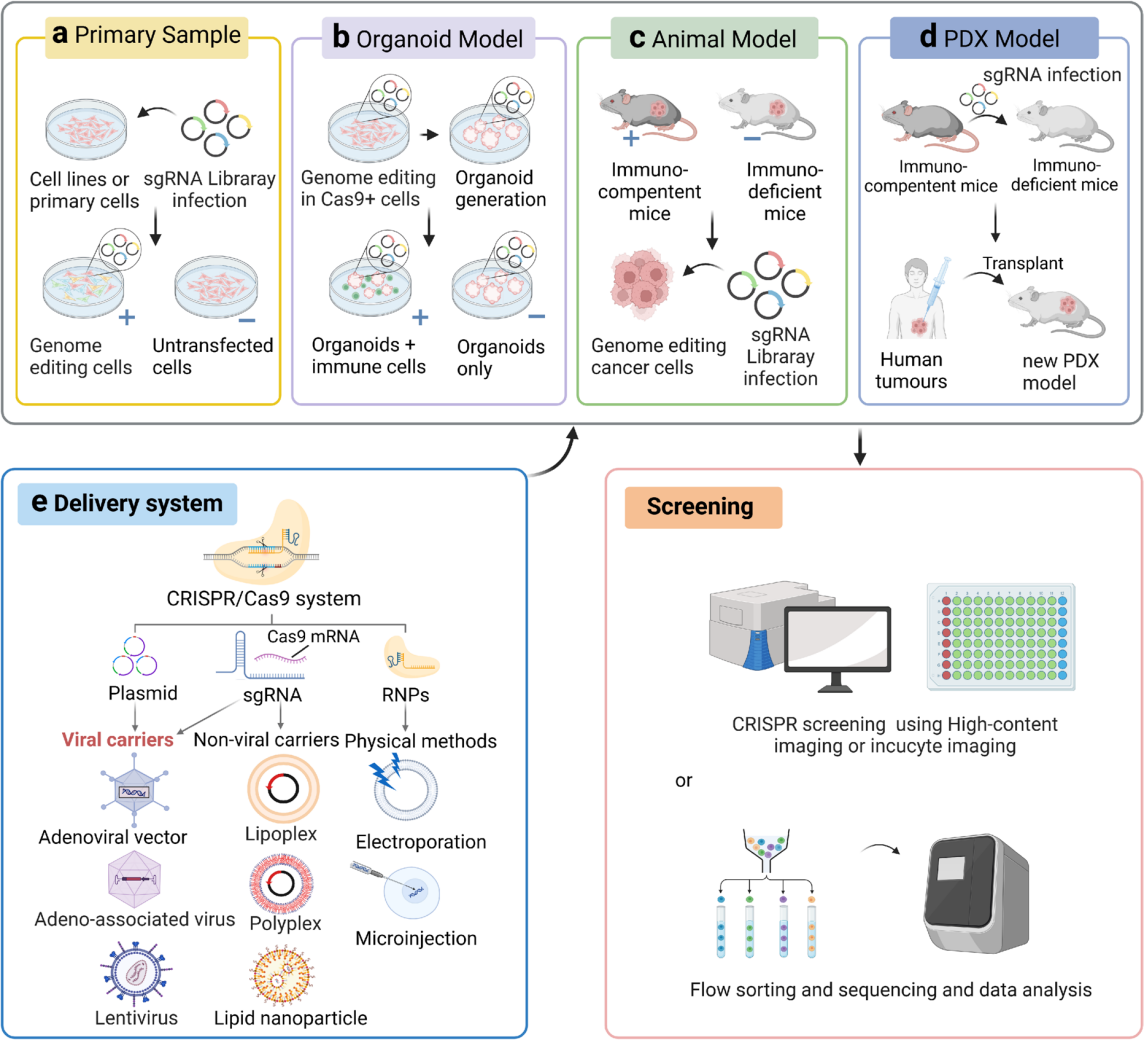
Preclinical screening models for CRISPR/Cas9. **a** Primary samples of transfected (experimental group) and untransfected (control group) CRISPR/Cas9 were screened and sequenced; **b** CRISPR/Cas9 in vitro screening were performed using organoids in a combined experiment; **c** CRISPR/Cas9 in vivo screening using immunodeficient mouse models; **d** CRISPR/Cas9 for generating new PDX mouse models. The immunocompetent and immunodeficient mice used are the same as those in (**c**); **e** Delivery forms of the CRISPR/Cas9 system includes delivery of plasmid, mRNA encoding Cas9, and RNPs. The delivery vectors encompass a diverse range of options, including viral and non-viral vectors, as well as physical methods of introduction

CRISPR/Cas9 technology plays a key role in modern biomedical research and is an effective tool for animal disease modelling. Its accuracy, efficiency and flexibility allow scientists to precisely introduce, alter or delete specific genes in the animal genome to create animal models that mimic the human tumour microenvironment. In vivo screening of CRISPR/Cas9 can restore the characteristics of environmental components, such as target cells, effector cells, immune modulating cells and extracellular matrix. Large genome-wide sgRNA libraries widely used mouse cells further demonstrate the effectiveness of CRISPR/Cas9 as a loss-of-function screening method and open up new avenues of research in fields such as immuno-oncology [62]. In contrast, in vitro CRISPR screening models are typically performed under cell culture conditions, allowing knockout or knock-in experiments to be performed on specific cell lines (Fig. 3a). In vitro screening allows rapid, high-throughput analysis of a large number of genes and is highly effective for initial characterization of gene function and screening for potential drug targets. However, this model may not be able to fully replicate in vivo conditions due to the lack of complexity in the physiological environment.

Organoids are in vitro 3D micro-organs that are cultured using small amounts of tissue and embryonic stem cells or induced pluripotent stem cells. They can be genetically encoded using CRISPR/Cas9 technology, allowing for the modeling of disease processes. Immune tumour genes can be screened and edited in organoids, providing new modelling directions for future clinical cancer research (Fig. 3b). Modelling organoid and T-cell co-cultures enables in vitro studies of immunosuppressive TMEs, to aid gene-targeted T-cell immunotherapy. After targeted knockdown of four breast cancer oncogenes (P53, PTEN, NF1 and RB1) in a breast cancer organoid model, the researchers observed an increase in tumourigenicity (Dekkers et al. 2020). The researchers conducted a CRISPR/Cas9 knockout of TP53, NF1, PTEN, SMAD4 and BAP1 genes in cholangiocyte organelles, their finding revealed that suppression of the BAP1 gene expression affected epithelial integrity in mice [63]. The development of a genome-wide CRISPR high-throughput screening platform has the potential to be applied in organoid models to screen genes for positive and negative survival [64]. A genome-wide CRISPR library forward screening approach was employed to identify genes associated with TGF-β resistance in WT, APC-KO, and KRASG12D mutant intestinal organs [65, 66]. The application of genome-wide CRISPR libraries to organoid tissues has been revolutionised to improve the accuracy and robustness of pooled-library CRISPR screens, while reducing the number of the cells required for genomic screening.

In 2013, Feng Zhang applied Cas9 technology to mammalian cells for the first time to establish disease models in mice [24]. Zhou Qi's team used the CRISPR-Cas system to induce Tet1/Tet2/Tet3 knockout in rats, achieving the first simultaneous multi-gene knockout in rats [67]. Jaenisch and his team at the Whitehead Institute worked to create a transgenic model of conditional knockout in mice, by piezo-driven injection of nucleic acids into the cytoplasm of mouse embryos [68]. The combination of CRISPR/Cas9 technology with animal disease models provides a powerful platform for disease mechanism research and drug screening development [69] (Fig. 3c). Martin et al.'s study revealed the mechanism of tumour suppressor gene (TSG) evolution under adaptive immune stress by applying a CRISPR screen in a mouse tumour model, whose deletion significantly increased the number of tumours. This finding highlights the process of clonal selection promoted by tumours by avoiding adaptive immune responses [70].

The construction of CRISPR/Cas9 knockout mouse models involves transfecting cell lines and establishing gene modification models. Among them, transfected cell lines are usually constructed using mouse embryonic stem cell culture or in vivo transplantation strategies, while gene modification models can be constructed with the assistance of embryonic stem cell-related techniques or by directly microinjecting CRISPR/Cas9-related factors into fertilized eggs. Establishing an animal model of small cell lung cancer (SCLC) through the Trp53 and Rb1 double knockout model, scientists have found that its pathogenesis is the deletion of p107 and p130 genes in carcinogenesis, which leads to accelerated tumourigenesis and spread [71]. These findings help the investigation of disease mechanisms and the development of new therapeutic targets in small cell lung cancer.

Tumour xenografts simulate tumour status more realistically than cell culture dish models, which fail to restore the tumour microenvironment or accurately respond to cellular load. The TME, which incorporates in situ cell implantation within the tumour, is a more suitable experimental model for data analysis. Patient-derived xenograft (PDX) models and patient-derived organoids-based xenograft (PDOX) models were constructed by implanting tumour cells and tissues extracted from patients after gene editing into immunodeficient mice (Fig. 3d). The combination of the PDX disease modelling with the CRISPR screening platform provides researchers with a valuable tool to study tumour mechanisms and drug screening [72]. He et al. constructed a rat model of PDX for squamous lung carcinoma with Rag1, Rag2, and Il2rg knockout [73]. The scientists employed this model for an in vivo CRISPR/Cas9 screen and identified an important metalloproteinase, a disintegrin and metalloproteinase domain-containing protein 10 (ADAM10). It is characterized by increased expression in slowly proliferating, treatment-resistant leukaemia stem cells. ADAM10 is an essential vulnerability factor in the pathogenesis of acute leukaemia and may serve as a therapeutic target to enhance the effect of conventional chemotherapy on leukaemia [74]. In another study, the PRMT5 gene was identified as a potential drug target in an in vivo CRISPR screen using a PDOX model. PRMT5 inhibitors synergistically enhanced gemcitabine's efficacy in the treatment of pancreatic cancer [75, 76].

#### The delivery systems of CRISPR/Cas9 technology

The delivery systems of CRISPR/Cas9 technology still face challenges in meeting the requisite clinical standards. Delivery systems must be efficient, safe and precise with appropriate controls in place, in order for them to be suitable for clinical treatment [77]. The successful delivery of CRISPR/Cas9 systems to specific target tissues or cell types is a major challenge, especially for tissues that are difficult to access, such as the brain or heart. CRISPR/Cas9 systems need to penetrate efficiently deep into the nucleus of the target cell, adding to the complexity of delivery. Currently, researchers are developing and testing a variety of ex vivo delivery methods, including viral vectors (e.g., lentiviruses [78] and adeno-associated viruses [79]), non-viral vectors (e.g., nanoparticles [80] and liposomes [81]), and physical methods (e.g., electroporation [82] and microinjection [83]). CRISPR/Cas9 systems are typically delivered using plasmids, viral vectors, or ribonucleoprotein particle (RNP) complexes (Fig. 3e). Ideal delivery systems should allow temporal control of CRISPR/Cas9 activity to prevent ongoing gene editing activities. Accurate control of the dose and activity of the CRISPR/Cas9 system in vivo is critical to ensure effective and precise gene editing. The off-target effects of CRISPR/Cas9 may trigger gene editing at non-target sites, which could lead to unexpected genetic changes and possibly even pathogenic mutations. At the same time, in vivo delivery may trigger an immune response, especially when using adenovirus (AdV) vectors. Adeno-associated virus (AAV) delivers and persists plasmid DNA containing CRISPR / Cas9 components into target cells, which leads to an increased chance of off-target activity [84]. This problem can be attenuated by using preassembled Cas and gRNA RNP complexes.

Furthermore, off-target effects are also reduced because AdV delivery vectors can integrate into the target cell genome. Lentiviruses (LVs) have many advantages as delivery vectors, but the integrative and persistent nature of transgene expression makes them unsuitable for CRISPR/Cas9 delivery. This is because random integration of viral DNA may result in activation or repression of cancer-related genes. The delivery of Cas9 genes via the self-assembling virus-like particle system results in transient expression, reducing the risk of off-targeting and eliciting an immune response [78]. Lipid-based nanoparticles (LBNPs) are a promising vector for delivery of the CRISPR/Cas9 system. They are commonly used for nucleic acid delivery loaded within cationic liposomes, which can easily cross the cell membrane of target cells. Cationic lipid-based transfection reagents such as liposomal amines are widely used for in vitro cell transfection, but they cannot be used in vivo due to their high cytotoxicity and interaction with anionic cell membranes, which impedes transfection efficiency in vivo tissues. This has limited the development of lipid carriers as a safe and efficient delivery system. A significant challenge in LBNP nanoparticle delivery is their accumulation in the liver and spleen. The presence of natural barriers at the delivery site, such as pulmonary fibrosis and the blood–brain barrier in the brain, further complicates the process. The delivery of RNP complexes via electrotransfection and microinjection has been demonstrated to be an effective approach for in vitro applications, with a higher editing efficiency and a reduced incident of fewer off-target mutations observed in electrotransfection compared to plasmid DNA transfection [85]. One limitation of microinjection is the necessity for specialized equipment and technologies to prevent damage to cells. Since only one cell is targeted per injection, the technique is not convenient when a large number of cells need to be processed. Søndergaard et al. demonstrated that the transfection efficiency and safety of human cancer cell lines were improved when large and small vectors were co-electrotransfected [86]. Patients with haematological diseases and cancer can already benefit from the clinical application of ex vivo gene therapy. However, in vivo delivery systems also face the challenge of degradation by proteases or nucleases or phagocytosis by monocytes [87]. The delivery strategies for the CRISPR/Cas9 system are still in need of refinement. There is an urgent need for safe and effective clinical delivery methods for patients, while avoiding immunogenicity and genotoxicity risks. There are also problems with addressing impeded delivery of large vectors, CRISPR toxicity, and inefficient DSB repair [88].

#### Case studies of CRISPR/Cas9 knockouts in clinical trials or translational research

The existing clinical trials employing the CRISPR/Cas9 technology have gradually progressed to the early clinical phases such as Phase I or II (Table 2). The optimization of engineered T cell efficacy using CRISPR/Cas9 technology has emerged as a prominent focus for clinical trials in recent years. PD-1 knockdown has been demonstrated to enhance the anti-tumour activity of T cells and improve the immunosuppressive environment in tumours [89]. In an FDA-approved CRISPR clinical trial in the United States, Stadtmauer et al., used CRISPR/Cas9 engineering of T cells to investigate immunotherapeutic treatments for refractory cancers [90]. The team knocked out the endogenous TCR genes TRAC and TRBC, and the PD-1 coding gene PDCD1. They also used a lentiviral vector to express a TCR specific for the NY-ESO-1 antigen in the engineered T cells, which were returned to patients for therapeutic study.

**Table 2:** Registered clinical trials of cancer immunotherapy edited using CRISPR/Cas9 technology

Mechanism	Target Site	Cell Type	Clinical Trial number	Condition or Diseases	Phase	Publications
Screening/knockout of immune modulatory Genes	CISH	TILs	NCT03057912	Human Papillomavirus-Related Malignant Neoplasm	Phase 1	[112]
			NCT04426669	Gastrointestinal Cancer	Phase 1/2	[113]
	NF1	iPSC	NCT03332030	Tumours of the Central Nervous System	Observational	[114]
	TP53	Patient Cancer Cells	NCT03606486	High-Grade Ovarian Serous Adenocarcinoma	Not Applicable	[115]
Optimisation of Immune Cell Function	CD70	Allogeneic CRISPR/Cas9-engineered T cells (CTX130)	NCT04502446	T Cell Lymphoma	Phase 1	[116]
			NCT04438083	Renal Cell Carcinoma	Phase 1	[117]
	CD25/TCR	CD19-specific CAR-T cells	NCT04557436	B Acute Lymphoblastic Leukemia	Phase 1	[118]
	CD19/CD20	anti-CD19/20-CAR vector-transduced T cells	NCT03097770	B Cell Leukemia; B Cell Lymphoma	Phase 1/2	[119]
	CD19/CD20/CD22	Universal Dual Specificity CD19 and CD20 or CD22 CAR-T Cells	NCT03398967	B Cell Leukemia; B Cell Lymphoma	Phase 1/2	[120]
	CD19	CRISPR-Edited Allogeneic Anti-CD19 CAR-T Cell (CB-010)	NCT04637763	Relapsed/Refractory B Cell Non-Hodgkin Lymphoma	Phase 1	[121]
	CD19	Allogeneic CRISPR/Cas9-Engineered T Cells (CTX110)	NCT04035434	B Cell Leukemia; B Cell Lymphoma	Phase 1/2	[122]
	Unknown	WT1-directed TCR T cells	NCT05066165	Acute Myeloid Leukemia	Phase 1/2	[123]
	BCMA	Allogeneic CRISPR/Cas9-Engineered T Cells (CTX120)	NCT04244656	Multiple Myeloma	Phase 1	[124]
	CD5	CT125A cells	NCT04767308	CD5 + Relapsed/Refractory Hematopoietic Malignancies	Early Phase 1	[125]
	B2M,/CIITA/TCR	CD19-specific CAR-T cells	NCT05037669	Acute Lymphoblastic; Leukemia; Non-Hodgkin Lymphoma	Phase 1	Not provided
	TCR/B2M	UCART019	NCT03166878	B Cell Leukemia; B Cell Lymphoma	Phase 1/2	[126]
		UCART019	NCT03229876	Acute Lymphoblastic Leukemia; Non Hodgkin Lymphoma (NHL)	Not Applicable	Not provided
	CD19	Mesothelin-directed CAR-T cells	NCT03747965	Solid Tumour	Phase 1	[127]
	CD7/TCR	CD7 UCAR-T cells	NCT04264078	Cell Leukemia; U-T-cell Lymphoma	Early Phase 1	[128]
Blocking Immune Checkpoints	TCR/PD-1	Anti-mesothelin CAR-T cells	NCT03545815	Solid Tumour	Phase 1	[95]
		NY-ESO-1 redirected autologous T cells	NCT03399448	Multiple Myeloma; Melanoma; Synovial Sarcoma;Myxoid /Round Cell Liposarcoma	Phase 1	[90]
	PD-1	Primary T-cells	NCT02793856	Metastatic Non-small Cell Lung Cancer	Phase 1	[129]
			NCT02863913	Invasive Bladder Cancer Stage IV	Phase 1	[130]
			NCT02867332	Metastatic Renal Cell Carcinoma	Phase 1	[131]
			NCT03525652	Prostate Cancer	Phase 1/2	Not provided
			NCT04417764	Advanced Hepatocellular Carcinoma	Phase 1	[132]
		PD-1 Knockout T Cells	NCT03081715	Tumours of the Central Nervous System	Not Applicable	[133]
		EBV-CTL cells	NCT03044743	EBV (Epstein-Barr virus) Positive Advanced-stage Malignancies	Phase 1/2	[134]
Disruption of Immune Evasion Mechanism Genes	TGFβR	CAR-EGFR T cells	NCT04976218	Advanced Biliary; Tract Cancer	Phase 1	[135]
	HPK1	XYF19 CAR-T cells	NCT04037566	Acute Lymphoblastic Leukaemia; Refractory Lymphoma	Phase 1	[136]
Enhancement of Antigen Presentation	TCR/HLA-I/ HLA-II	PACE CAR-T19 cells	NCT05037669	Acute and Chronic Lymphoblastic Leukemia; Non Hodgkin Lymphoma	Phase 1	[137]

The data were analysed using the ClinicalTrials.gov database

CAR-T cells with CRISPR/Cas9-mediated PD-1 editing technology have been clinically tested in a variety of cancer models. These include pairs of adult human mesothelin-directed CAR-T cells and mesothelin-resistant CAR-T cells in solid tumours, as well as CAR-T cells in chronic lymphocytic leukaemia, T cell lymphoma and B-cell lymphoma. In conclusion, CRISPR technology for CAR-T cells for tumour immunotherapy is gradually entering clinical trials. PACE CAR-T19 are precision engineered allogeneic cell therapies that not only target CD19, but also interfere with beta-2 microglobulin (B2M), class II major histocompatibility complex activator (CIITA) via CRISPR/Cas9 and TCR-alpha chain (TRAC), aiming to reduce the immune response and increase the effectiveness of cell therapy in patients. In 2022, scientists published the results of a Phase I clinical trial for the treatment of aggressive non-Hodgkin's lymphoma using PACE CAR-T19 cells with a knockout of the PD-1 gene, and the treatment was generally well tolerated with an acceptable safety profile.

In addition, based on the success of CAR-T cell therapies in the treatment of leukaemia and lymphoma, CRISPR Therapeutics incorporation has developed allogeneic CAR-T cell therapies for CTX110, CTX120 and CTX130 and has conducted Phase 1 clinical trials for the treatment of various types of cancer. Each of these therapies employs CRISPR/Cas9 gene editing technology to optimise the ability of a patient's T cells to fight cancer. CTX110 has been modified with CRISPR/Cas9 technology to target CD19-positive B-cell malignancies. CTX120 targets the B-cell maturation antigen (BCMA) and is primarily used to attack BCMA-expressing multiple myeloma. CTX130 is an anti-CD70 CAR-T cell therapy for the treatment of solid tumours such as renal cell carcinoma and T-cell lymphomas.

CRISPR/Cas9 also screens and knocks out genes related to immune regulation, enhancing the anti-tumour capacity of immune cells. It has been used to treat human papillomavirus-associated malignancies or gastrointestinal cancers by targeting the CISH gene in TLC cells. Scientists have also developed Crispr-Duplex sequencing technology to detect tumour-associated mutations in TP53 (the most frequently mutated gene in ovarian cancer) in these samples, which has great potential for advances in cancer diagnostic tools.

#### Assessing the efficacy and safety of CRISPR/Cas9 knockouts in *cancer* immunotherapy

While CRISPR-assisted cancer immunotherapy holds promise for patients, there are still some barriers to clinical translation, such as immunogenicity challenges. A fatal severe immune reaction event occurred in 1999 [91], when Jesse died of a severe immune reaction after being injected with an adenovirus encoding the OTC gene, the first recorded death related to gene therapy in a clinical trial. The U.S. Food and Drug Administration (FDA) recently issued a decision on a draft Investigational New Drug (IND), emphasizing that ensuring the safety of CRISPR in combination with immunotherapy is a top priority for clinical translation [92]. Several clinical trials conducted by scientists have confirmed the clinical feasibility and safety of CRISPR/ Cas9 modification of T cells. Lu's team treated 12 patients with non-small cell lung cancer with PD-1-edited T cells (NCT02793856). In this clinical trial, peripheral blood lymphocytes were collected from patients and the PDCD1 gene was knocked out using CRISPR/Cas9 technology in a laboratory setting. The screened cells are injected back into the patient. The primary purpose of the study was to evaluate the safety and side effects of the treatment, not to evaluate its efficacy. In 2022, a recent CRISPR clinical trial reported some side effects, including fever, rash, and fatigue during treatment, as well as poor editing efficiency of T cells injected back into patients, with only a median of 6% being edited. Edited T cells were detected in 11 patients after two months, although at low levels [93]. This preliminarily suggests that the treatment is safe and feasible with mild side effects. In 2020, Stadtmauer's study [90] showed that edited T cells steadily infiltrate and implant into tumour areas after treatment. This also indicates that the treatment is safe and well tolerated by patients, and the incidence of adverse reactions is within the acceptable range. These findings provide preliminary evidence for the safety and feasibility of CRISPR/Cas9 gene editing on modified immune cells in a clinical setting. In a subsequent phase I clinical trial, Lacey and Fraietta [94] implanted edited T cells into patients with advanced NSCLC, and Wang et al. [95] injected PD-1 gene-edited mesotheliin-specific CAR T cells (MPTK-CAR T cells) into patients with solid tumours. All these clinical trials have come to the same conclusion.

### Challenges and future directions

#### Potential synergistic approaches to combining CRISPR/Cas9 knockouts with other immunotherapies

**Fig. 4:**
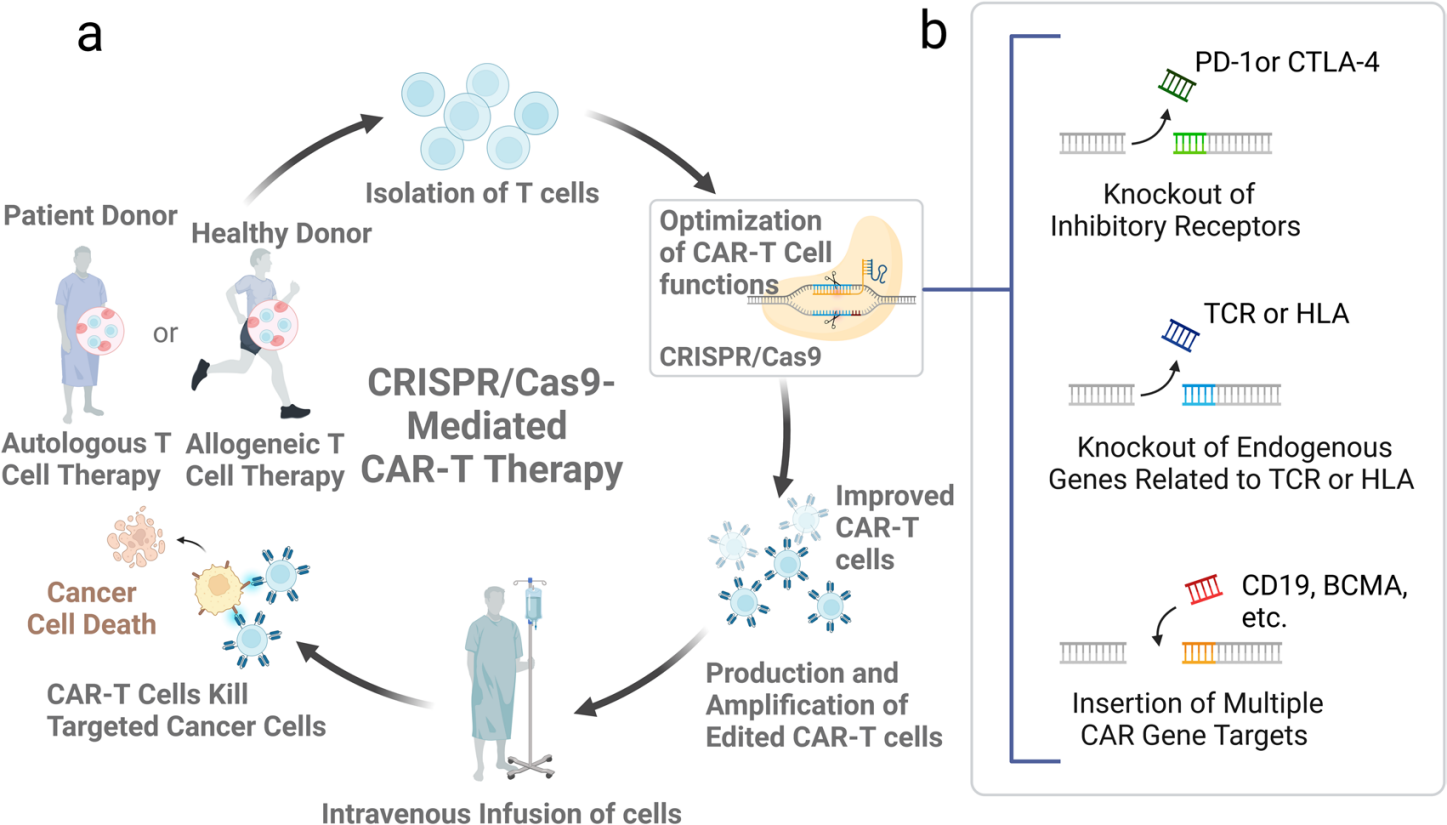
Technical flow of autologous CAR-T cell therapies and allogeneic CAR-T cell therapies combined with CRISPR/Cas9 technology. **a** Technical workflow of CRISPR/Cas9 genome editing technology employed to facilitate autologous CAR-T cell therapy and allogeneic CAR-T cell therapy; **b** CRISPR/Cas9 modifies CAR-T cells in various manners, such as disrupting genes associated with immune checkpoints and antigen presentation, as well as introducing multiple CAR genes into CAR-T cells that target diverse cancer cell antigens

At present, the advanced synergistic therapies for CRISPR/Cas9 are the optimisation of CAR-T cell therapies and the development of allogeneic CAR-T cell therapies. Clinical trials have demonstrated that the therapeutic potential and efficacy of CAR-T cell therapies for a wide range of haematological and solid tumours, but they still confronted with significant challenges, including immunosuppressive TME, antigen loss and CAR-T cell depletion, in a multitude of applications [96]. The ability of T cells to eradicate cancer cells is stimulated after precise genetic modification of CAR-T cells using CRISPR/Cas9 technology (Fig. 4a). This offers new hope for cancer patients by effectively mitigating immune rejection and side effects, optimizing the function of CAR-T cells on multiple levels and overcoming challenges such as the limited availability of target antigens and problems with the tolerability of therapeutic effects.

There are two types of customized genetic CAR-T cell therapies that show great potential in cancer immunotherapy, one is autologous CAR-T cell therapy and the other is allogeneic CAR-T cell therapy. For autologous T cell therapy, T cells are collected from the patient's own blood. After being genetically edited in vitro, they are made to express specific chimeric antigen receptors. These receptors are capable of recognizing and binding to specific antigens on cancer cells. Modified CAR-T cells are cultured and proliferated before being re-infused back into the patient via intravenous injection. Using CRISPR/Cas9 technology, genes or inhibitory receptors (e.g., PD-1 or CTLA-4) for endogenous TCR and HLA can be knocked down in T cells, and multiple targets of CAR-T cells such as CD19, BCMA, CD22, and CD20 can be edited in parallel [97] (Fig. 4b). Enhanced CAR-T cell specificity for targeted attack on cancer cells may lead to increased persistence and anti-tumour activity of CAR-T cells, as well as improved safety of precision therapy. For example, the potential side effects of CAR-T cell therapy could be reduced by precisely controlling CAR expression levels or introducing safety switches in studies of cytokine release syndrome (CRS) [90]. In a mouse model of acute lymphoblastic leukaemia xenograft, CRISPR/Cas9 technology was able to precisely edit immune cells to optimise CAR-T cells targeting CD19 [98]. These cells showed significantly improved efficacy of cell therapies for leukaemia. Choi employed CRISPR multigene editing technology to knock out the endogenous TRAC, B2M, and PD-1, rendering CAR-T cells resistant to PD-1 inhibition [61]. In preclinical glioma models, these triple gene-edited CAR-T cells showed enhanced activity. The advantage of autologous CAR-T cell therapy is that immune rejection can be reduced by using the patient's own cells. For example, CD19-specific CAR-T cells (UCART019) can prevent graft-versus-host disease (GVHD) and minimize its immunogenicity. While CAR-T cell therapy has shown promising results in the treatment of B cell lymphomas and leukaemia, there are challenges that limit its broad application. The manufacturing process is intricate, time-consuming, and expensive, which can make it inaccessible to many patients. Additionally, there are instances where the treatment may not be effective, or the patient may be unable to tolerate the high levels of treatment intensity. Allogeneic CAR-T therapy differs in that it harvests T cells from healthy donors. These donor T cells are then genetically modified to express specific chimeric antigen receptors and cultured to express specific CARs. Finally, these generic CAR-T cell products are administered intravenously to the patient. One of the main benefits of this approach is that it provides a ‘ready-to-use’ and rapid treatment option that does not require a time-consuming wait for T cells to be harvested and modified from the patients. However, the use of foreign T cells also increases the risk of complications from immune rejection, such as GVHD, compared to autologous therapy [99].

In 2022, CRISPR Therapeutics incorporation published the results of their Phase I clinical trial in the United States of two allogeneic CAR-T therapies targeting CD70 for the treatment of certain lymphomas and certain solid tumours, as well as CD19 for the treatment of leukaemia and lymphoma. The results showed a favorable safety and tolerability profile, with minimal adverse effects and a high safety profile. Overall, both strategies continue to innovate with improved efficacy and safety, and reduced risk and cost. CRISPR-mediated CAR-T therapy is a highly personalised, precise, and efficient strategy for cancer treatment. It promises to dramatically increase treatment success rates and bring more hope to patients.

#### Challenges in the application of CRISPR/Cas9 technology in vivo

The in vivo application of CRISPR/Cas9 technology still faces a number of challenges that must be addressed if the technology is to realize its full potential in clinical therapeutics. Off-target effects of the CRISPR/Cas9 system can lead to mutations that do not match expectations and increase safety risks in clinical therapies. CRSIPOR, Cas-OFFinder, and CasOT are tools or algorithms that are used to predict or detect off-target effects [103]. To circumvent off-target effects, scientists have developed Cas9 variants that reduce such effects, including enhanced eSpCas9, SpCas9-HF1, and hypaCas9. Hyperprecise hypaCas9 exhibits higher on-target activity compared to the earlier variants, eSpCas9 and SpCas9-HF1 [104]. The use of Cas protein homologs with the ability to recognize longer sequences or rarer PAM sequences also improves the editing specificity of target genes [105]. Alternatively, modification of SgRNA sequences can improve the specificity of recognition. Finally, AcrIIA4 (anti-CRISPR protein) has also been shown in studies to reduce off-target effects [106].

In addition, CRISPR/Cas9 lacks a safe and effective delivery system that meets the standards required for clinical applications of gene editing [77]. Currently, a relatively novel CRISPR/Cas9-mediated gene editing system employs polyethyleneimine (PEI) magnetic nanoparticles (MNPs) as a gene delivery method. This nontoxic strategy has little nonspecific effect on other cells [86]. However, electroporation and viral vectors remain broader options. Research on CRISPR delivery strategies requires intensive investigation and continuous improvement. Furthermore, future problems such as blocked delivery of large vectors, development of low-immunogenic Cas protein variants, and inefficient DSB repair must be addressed. Although many algorithms and experimental methods designed to predict and minimise off-target effects. the complete elimination of these issues remains a challenge.

These issues can be addressed with advanced CRISPR tools such as CRISPR/Cas12a. Cas12a is the other endonuclease in the CRISPR family for genome editing. Cas12a has unique advantages over Cas9 in tumour immunotherapy. Cas12a simplifies the process of processing CRISPR arrays by splitting them into individual crRNAs. Cas12a can simultaneously edit multiple gene targets by expressing multiple crRNAs from a single transcript [107]. Nazanin et al. used Cas12a to construct an innovative human gene library, designated as the ‘in4mer’ platform. The ability of Cas12a to independently target up to four genes makes the construction of multiple gene editing libraries more efficient. Additionally, it is smaller than CRISPR/Cas9 libraries and possesses the capacity to detect complex gene interactions [108]. Dai et al. developed the modular AAV-Cp9 library. modular AAV-Cpf1 KIKO system, which combines CRISPR-Cpf1 and AAV. This technology surpassed the sevenfold efficiency of CRISPR/Cas9 knock-in, enabling more efficient gene double knock-in in CAR-T cells [109]. Sidi Chen et al. developed the Cas12a/Cpf1 mRNA with AAV CLASH system in combination with Cas12a/Cpf1 mRNA and AAV, aiming to overcome the low efficiency and limited scale of targeted gene knock-in in cell therapy. In practice, CLASH promotes the proliferative capacity and efficacy of CAR-T cell variants in blood and solid tumour models, which include the CD19 + CD22 + NALM6 tumour model and HER2 + HT29 tumour model [110]. These modular systems feature multiple knock-ins as well as the ability to optimise CAR-T, marking an advance in the development of more effective tumour cell therapies. The researchers successfully delivered the CRISPR-Cas12a system with an oncolytic adenovirus (oAd) vector in vivo, enabling gene editing targeting the epidermal growth factor receptor (EGFR). This significantly induced apoptosis in tumour cells, ultimately leading to complete tumour regression in some of the treated mice. The system has the potential to assist in tumour immunotherapy [111]. CRISPR/Cas12a technology has the potential to dramatically enhance the function of CAR-T cells in complex tumour microenvironments. In the future, the technology is expected to improve the specificity and efficacy of immunotherapy and overcome the limitations faced by CRISPR/Cas9.

#### Future developments and applications of CRISPR/Cas9 technology in tumour immunotherapy

In future iterations of knockout technology, the integration of CRISPR technology with spatial genomics holds significant potential. Dhainaut created a spatial genomics platform called Perturb-map to investigate regulatory variables in the tumour microenvironment using innovative technology [100]. This platform surpasses the limitations of conventional CRISPR screening methods by effectively detecting the roles of genes outside of cells in the tissue environment. In addition, it can determine the localization of specific genomic DNA within the nucleus. Scientists used the Perturb-map platform to clarify the function of interfering genes in lung cancer. This was achieved by simultaneously disabling numerous genes in a mouse model of lung cancer and conducting a comprehensive analysis of phenotypes using imaging and spatial transcriptomics. This study found that when the Tgfbr2 gene, which is responsible for tumour growth, was suppressed in lung cancer cells, it led to the restructuring of the tumour microenvironment and enhanced tumour immune response. This indicates that using spatial CRISPR genomics techniques can uncover regulators within the tumour microenvironment and present novel potential targets for future cancer treatments.

Nanotechnology has significantly influenced the spatiotemporal processing of the CRISPR gene editing process, resulting in reduced harmful effects on genes. Surface-modified nanocarriers are capable of traversing biological membranes and enhancing the effectiveness of delivering substances within cells [101]. Compared to viral vectors, nanocarriers present a reduced safety hazard and generally exhibit less immunogenicity and pathogenicity. Magnetic nanocarriers can be precisely manipulated using external controls, such as magnetic fields, to achieve spatial control over CRISPR/Cas9 editing activities. This allows precise control of the location of gene editing. Zhu et al. examined the effectiveness of a novel magnetic nanocarrier-based complex, known as the MNP-BV complex, in facilitating CRISPR/Cas9 editing [102]. The manipulation of gene editing in living organisms was accomplished using a magnetic field. The virus vector used is a baculovirus (BV) that infects insects and has a high capacity for packaging genetic material (> 38 kb). This vector is able to transfer and express genes in mammalian cells without integrating into the host genome. However, BV is rendered inactive by the serum complement system when delivered in vivo. The activation of BV transduction in vivo can be achieved by forming MNP-BV complexes by attaching magnetic nanoparticles (MNPs) to BV. This activation occurs specifically in the presence of an applied magnetic field. The MNP-BV complexes demonstrate exceptional efficacy in converting magnetic fields into BV transduction and gene expression, enabling precise gene editing in various tumour locations. While nanocarriers are now inadequate for widespread clinical use, advances in biomaterials can help expand the scope of gene editing tools.

## Conclusion

The precision of CRISPR/Cas9 gene editing technology in editing the genomes of immune cells or tumour cells has been proven to be a powerful tool for tumour immunotherapy. Wide-ranging gene screening and modification using CRISPR/Cas9 technology can deeply explore tumour immunotherapy targets from a genetic level. At the same time, it can also help improve the defects of CAR-T cell therapy, such as T cell exhaustion, low production efficiency, and poor persistence in the body. However, there are some obstacles to applying CRISPR/Cas9 technology in clinical tumour immunotherapy, including ensuring the accuracy and safety of gene editing, preventing unexpected side effects, solving the difficulties of delivering CRISPR systems in vivo, and overcoming the obstacles brought by the diversity and complexity of tumours. The future direction of CRISPR/Cas9 research will still be to improve the effectiveness and safety of treatment, and to strive to customize personalized medical care based on the specific tumour characteristics of patients. As our understanding of the tumour microenvironment and immune evasion mechanisms deepens, it is expected that new treatment approaches and enhanced immunotherapy combinations will be developed, bringing greater hope to patients.

## Data Availability

All Figures were generated using the BioRender platform with the requisite permission for publication. No datasets were generated or analysed during the current study.

## Abbreviations

ADAR1: Adenosine Deaminase Acting on RNA 1
B2M: Beta-2-Microglobulin
CBLB: Casitas B-lineage Lymphoma B
CAR-T: Chimeric Antigen Receptor T-cell
Cas9: CRISPR-associated protein 9
CRISPR: Clustered Regularly Interspaced Short Palindromic Repeats
CRISPRa: CRISPR activation
CRISPRi: CRISPR interference
CTLA-4: Cytotoxic T-Lymphocyte Associated Protein 4
DHX37: DEAH-Box Helicase 37
DSB: Double Strand Break
dCas9: Dead Cas9 (catalytically inactive Cas9)
dsDNA: Double-stranded DNA
EGFRvIII: Epidermal Growth Factor Receptor variant III
FAK: Focal Adhesion Kinase
HDR: Homology-directed Repair
HLA: Human Leukocyte Antigen
ICI: Immune Checkpoint Inhibitor
IFN-γ: Interferon Gamma
JAK/STAT: Janus Kinase/Signal Transducer and Activator of Transcription
KRAS: Kirsten Rat Sarcoma Viral Oncogene Homolog
LCP2: Lymphocyte Cytosolic Protein 2
MHC-I: Major Histocompatibility Complex Class I
MR1: MHC Class I-related Protein 1
NHEJ: Non-Homologous End Joining
PAM: Protospacer Adjacent Motif
PD-1: Programmed Death-1
PD-L1: Programmed Death-Ligand 1
PTPN2: Protein Tyrosine Phosphatase Non-Receptor Type 2
RASA2: RAS p21 Protein Activator 2
RNP: Ribonucleoprotein
sgRNA: Single-guide RNA
SOCS1: Suppressor of Cytokine Signaling 1
SUSD6: Sushi Domain Containing 6
TALEN: Transcription Activator-Like Effector Nucleases
TAP1/TAP2: Transporter Associated with Antigen Processing 1 and 2
TCEB2: Transcription Elongation Factor B (SIII), Polypeptide 2
T-DM1: Trastuzumab emtansine
TMEM127: Transmembrane Protein 127
TILs: Tumour-Infiltrating Lymphocytes
tracrRNA: Trans-activating CRISPR RNA
WWP2: E3 Ubiquitin Protein Ligase WWP2
ZFN: Zinc Finger Nucleases
